# Production and characterization of breadfruit flour (*Artocarpus altilis*): an alternative for the elaboration of flakes for children’s alimentation

**DOI:** 10.3389/fnut.2025.1673509

**Published:** 2026-01-23

**Authors:** Cristina Arteaga, Ruth Arias-Gutiérrez, Adriana Rodríguez, Susana Heredia-Aguirre, Mónica Gozalbo, Jesús Blesa, Renata Alejandra Alvarado-Barba, Cristina Gabriela Ríos-Romero, Dayana Villavicencio Barriga, Jessenia Vásquez, Karol Egas, Elizabeth Quiroga-Torres, Alberto Bustillos, Tannia Valeria Carpio-Arias

**Affiliations:** 1 Carrera de Nutrición y Dietética, Facultad de Ciencias de la Salud, Universidad Técnica de Ambato, Ambato, Ecuador; 2 Facultad de Ciencias de la Vida, Universidad Estatal Amazónica, Puyo, Ecuador; 3 Faculty of Sciences, and Pharmacy Program, Riobamba-Chimborazo, Escuela Superior Politécnica de Chimborazo, Riobamba, Ecuador; 4 Carrera de Nutrición y Dietética, Facultad de Salud Pública, Escuela Superior Politécnica de Chimborazo, Riobamba, Ecuador; 5 Department of Preventive Medicine and Public Health, Food Sciences, Toxicology and Legal Medicine, Universitat de València, Valencia, Spain; 6 Sede Orellana, Escuela Superior Politécnica de Chimborazo, Riobamba, Ecuador; 7 Research Group on Human Food and Nutrition (GIANH), Riobamba-Chimborazo, Escuela Superior Politécnica de Chimborazo, Riobamba, Ecuador; 8 Ecuador Kuskiykuy Academic and Scientific Research Group in Biomedical Sciences with Social Projection, Yachay Suntur, Technical University of Ambato, Ambato, Ecuador; 9 Laboratorio de Bioquímica, Carrera de Agronomía, Facultad de Ciencias Agropecuarias, Universidad Técnica de Ambato, Ambato, Ecuador

**Keywords:** breadfruit flour, extrusion cooking, breakfast cereal flakes, sensory acceptability, food safety

## Abstract

**Introduction:**

Breadfruit (*Artocarpus altilis*) represents a promising tropical crop with high nutritional potential and application in functional food development. This study aimed to produce and characterize gluten-free breadfruit flour (BF) and evaluate its use at 20.7% substitution in corn-based extruded flakes.

**Methods:**

Physiologically mature breadfruit was peeled, cored, cut into 20 × 20 × 10 mm pulp cubes, dipped in 0.1% (w/v) sodium bisulfite for 2 min, tray-dried at 70°C (air velocity 1.5 m s^−1^), and milled to < 212 μm.

**Results:**

The resulting flour exhibited a moisture content of 10.8%, protein 10.1%, fat 2.4%, fiber 4.06%, and total phenolics of 298.7 mg GAE/100 g, with a predominance of polyunsaturated fatty acids (52.8%) and 41.4 mg β-carotene/100 g. Heavy metals (Pb = 0.085 mg/kg, Cd = 0.012 mg/kg) and aflatoxins (< 0.5 μg/kg) were below national safety limits, confirming product safety.

**Discussion:**

The optimized formulation (F3, 20.7% BF) was extruded under a temperature profile of 80/110/135°C and 200 rpm, producing flakes with favorable expansion (4.0 ± 0.1), hardness (14.7 ± 0.4 N), and high sensory acceptability (7.9 ± 0.33). All products complied with microbiological standards. The integration of BF improved the nutritional and functional value of the flakes, offering a sustainable and safe alternative ingredient for developing fortified breakfast cereals. These findings highlight breadfruit flour as a technologically feasible, nutrient-dense, and sustainable resource to enhance food diversification and support nutrition-sensitive strategies addressing malnutrition in Ecuador and other tropical regions.

## 1 Introduction

Breadfruit (Artocarpus altilis), also known as “fruto pan” or “frutepan,” is a high-yield food tree that produces fleshy, oval-shaped fruits that can reach up to 30 cm in length and 20 cm in diameter (1). The fruit’s weight varies depending on the variety, generally ranging between 1 and 5 kg. Its surface exhibits a rough texture, with a thick and hard skin that changes color from green to dark brown as it ripens (2). Originally from the Indomalaya biogeographic realm, breadfruit cultivation has expanded widely due to its nutritional qualities, now being present across the Oceanian, Neotropical, Afrotropical, and Australian realms. It thrives in various biomes, including tropical and subtropical moist broadleaf forests; tropical and subtropical grasslands, savannas, and shrublands; mangroves; Mediterranean forests, woodlands, and scrub; temperate broadleaf and mixed forests; temperate grasslands, savannas, and shrublands; and even deserts and xeric shrublands (1, 3).

In Ecuador, breadfruit is primarily cultivated in the Coastal and Amazon regions, particularly in the provinces of Manabí, Los Ríos, Guayas, and Esmeraldas. In the Amazon region, it is mainly grown in Napo and Sucumbíos. It is also cultivated in the Galápagos Islands and in the Andean region, notably in the provinces of Pichincha and Santo Domingo de los Tsáchilas. This plant adapts well to various soil types with good fertility and drainage and thrives in warm, humid climates with temperatures ranging from 20 to 35°C and annual precipitation between 1,000 and 3,000 mm. The tree can grow up to 25 m in height and begins to bear fruit 3–5 years after planting.

Breadfruit is valued both for its nutritional benefits and its culinary versatility (4). Among its most notable properties is its high fiber content, which promotes digestion and gastrointestinal health. It is also attributed a low glycemic index, which helps maintain stable blood sugar levels, and a richness in antioxidants that contribute to combating cellular damage and preventing chronic diseases such as cancer and heart disease (4, 5). Additionally, it is an important source of essential vitamins and minerals, including vitamin C, which strengthens the immune system; vitamin A, which supports visual health; potassium, which regulates blood pressure; calcium, which strengthens bones; and iron, which combats anemia and chronic malnutrition in children (6, 7). Given its nutritional profile, breadfruit shows great potential for use in the development of foods aimed at combating child malnutrition in Ecuador, a public health issue affecting a significant part of the population (6, 8). Its high nutrient density makes it an ideal ingredient for the development of fortified foods that can be incorporated into food assistance programs, particularly in rural communities and vulnerable areas. In the culinary field, breadfruit offers multiple applications (9, 10). It can be used in savory dishes, such as stews, soups, curries, and salads; in desserts, such as cakes, pies, and ice cream; and as a flour for producing gluten-free baked goods, including breads and cookies.

Although breadfruit industrialization in Ecuador is still in its early stages, efforts are underway to develop derivative products, such as breadfruit flour, which is used in the preparation of breads, cookies, and other baked or extruded foods. However, this food remains underutilized in the food industry, representing a significant opportunity for innovation in the development of new products and processes. Promoting research and the creation of breadfruit-based foods could open new horizons for its commercialization and mass consumption, fostering its recognition as a sustainable and nutritious food resource.

The objective of the present study is to characterize the flour obtained from Artocarpus altilis fruits, used in the production of flakes with three formulations of corn flour, through bromatological, microbiological, and sensory analysis.

## 2 Methods

### 2.1 Raw materials

Physiologically mature breadfruit was harvested in Manabí Province, Ecuador (0°58’ S, 80°17’ W), transported to the laboratory within 24 h, and inspected to remove fruits with mechanical or microbial damage. Commercial maize grits (≥98% purity), sucrose, malt extract, chocolate flavoring, and food-grade colorants were sourced locally. All reagents were of ACS analytical grade.

### 2.2 Production of breadfruit flour

The selected breadfruit was free from spots or signs of fungal contamination, ensuring only the best quality pulp was used. The seeds were roasted, peeled, and chopped into small pieces (approximately 20 × 20 × 10 mm). To prevent enzymatic browning, pulp pieces were immersed in a 0.1% (w/v) sodium bisulfite solution for 2 min, drained, and then tray-dried at 70 °C (with an air velocity of 1.5 m/s) until moisture content was reduced to ≤ 10% (wet basis). The dried pulp was ground, passing through a 212 μm sieve to obtain a fine powder resembling flour. This flour was then stored in laminated aluminum pouches for further use.

The quality control of the breadfruit flour followed the standards outlined in NTE INEN 616:2015 (Ecuadorian standard for wheat flour) and NTE INEN 2553:2014 (for food flour standards), while corn flour was controlled according to NTE INEN 2051:2013 (Ecuadorian standard for corn flour).

### 2.3 Composite-flour formulation

Three composite blends were prepared by pre-mixing breadfruit flour (BF) with varying proportions of maize grits, sucrose, malt extract, chocolate flavor, and colorant as needed (Table 1). The moisture content of the feed was adjusted to 22% using deionized water, and the mixture was equilibrated for 30 min at 25 °C to ensure uniformity before extrusion.

**TABLE 1:** Formulations for preparing breadfruit and corn flakes.

Ingredients	Formula 1 (F1)	Formula 2 (F2)	Formula 3 (F3)*
Corn flour (%)	62.11	52.11	54.03
Breadfruit flour (%)	20.70	20.70	20.70
Malt extract (%)	4.14	4.14	4.14
Chocolate flavoring (%)	0.62	3.62	3.62
Sugar (%)	12.42	17.42	16.00
Coloring agent (%)	0.00	2.00	1.50

*Formula 3 (F3) exhibited the best texture among the tested formulations and was selected for the final product development.

### 2.4 Extrusion cooking

The composite blends were processed in a pilot-scale single-screw extruder (Brabender KE-19, 19 mm Ø, L/D = 20) at a barrel temperature profile of 80/110/135 °C and a screw speed of 200 rpm. The die diameter was set to 3 mm, and the specific mechanical energy averaged 110 ± 5 kJ/kg. After extrusion, the product was knife-cut into 15 mm pieces, dried in a forced-air oven at 65 °C until the moisture content dropped to < 8%. The dried flakes were equilibrated for 6 h at ambient conditions and then sealed in metallized pouches to prevent moisture absorption.

**FIGURE 1:**
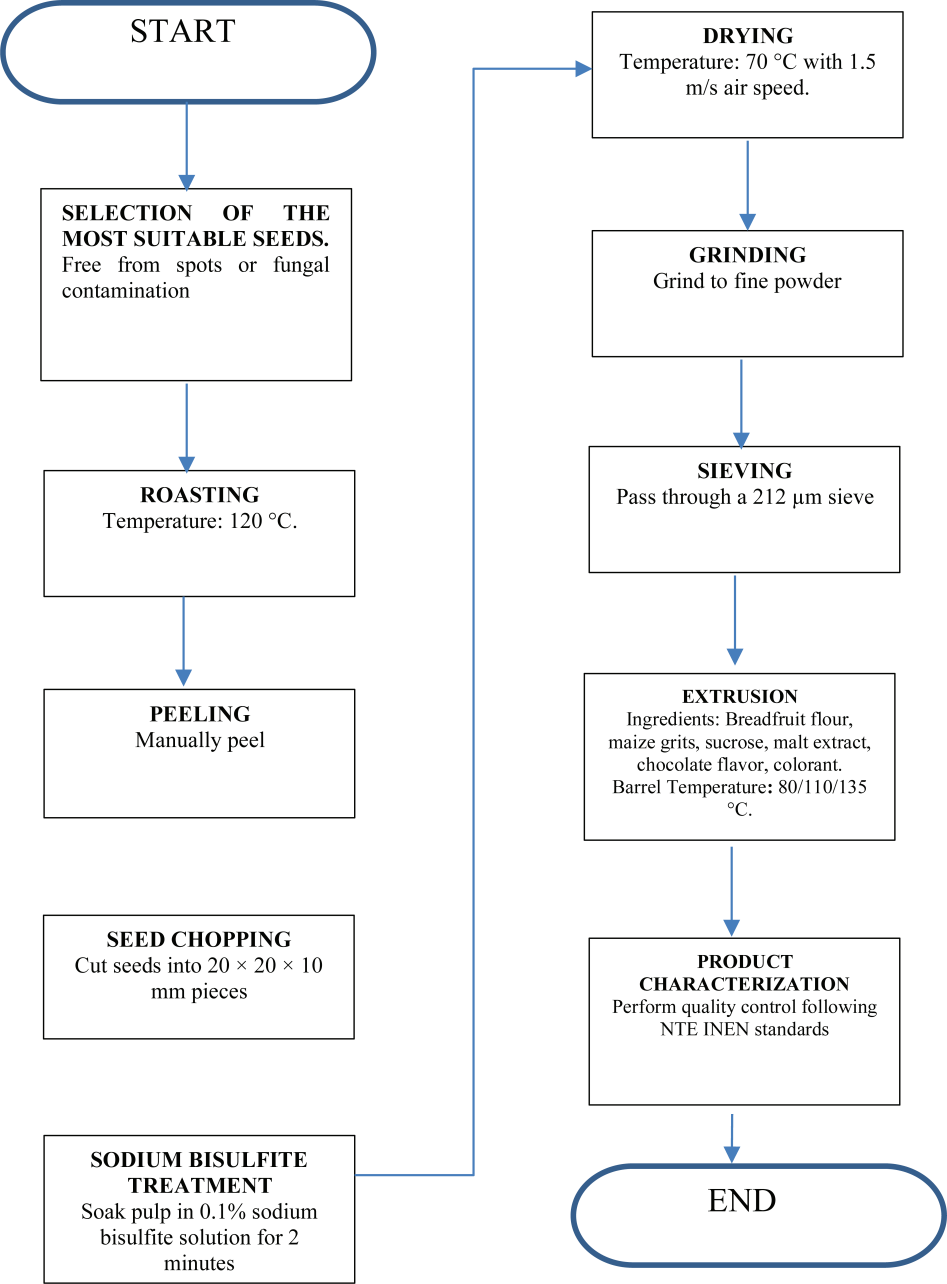
Process of obtaining breadfruit flour.

Figure 1 outlines the step-by-step process for producing breadfruit flour, including seed selection, roasting, peeling, chopping, bisulfite treatment, drying, grinding, sieving, and extrusion, with product characterization following NTE INEN standards.

### 2.5 Proximate and techno-functional analyses

Samples were analyzed in triplicate for moisture (AOAC 925.10), protein (Kjeldahl method, AOAC 2001.11), crude fat (Soxhlet extraction, AOAC 2003.06), ash (muffle furnace, AOAC 923.03), and total dietary fiber (AOAC 985.29). Additional analyses included titratable acidity (NTE INEN-ISO 521) and particle-size distribution (NTE INEN-ISO 517). Functional properties such as expansion ratio, bulk density, hardness (measured with a TA-XT2i texture analyzer, 3-point bend), and water absorption index (WAI) were determined using approved AACCI methods (American Association of Cereal Chemists International).

The total phenolic content was extracted using an ethanol-water (50:50 v/v) solution at 45 °C for 40 min and quantified by the Folin-Ciocalteu method (GAE, Gallic Acid Equivalents). Carotenoids were determined by HPLC-DAD (high-performance liquid chromatography with diode array detection) after acetone extraction. Fatty acid methyl esters were analyzed using gas chromatography with a flame ionization detector (GC-FID, Agilent 7890A, DB-23 column, 230 °C detector).

### 2.6 Microbiological and chemical safety

Microbiological analyses included molds and yeasts (NTE INEN 2561:2010), E. coli (microFAST, NTE INEN 2561:2010), and mesophilic aerobes (NTE INEN 540:2010), conducted on both raw materials, BF, and extruded flakes. All microbial counts were well below the national limits set by Ecuadorian regulations. Chemical contaminants, including lead (Pb), cadmium (Cd), and aflatoxins (B_1_, B_2_, G_1_, G_2_), were quantified. Lead and cadmium were determined using flame atomic absorption spectroscopy (PerkinElmer AAnalyst 400), and aflatoxins were detected by competitive enzyme-linked immunosorbent assay (ELISA, R-Biopharm kit).

### 2.7 Sensory evaluation

Sensory evaluation was performed with 100 untrained volunteer panelists (50% women, 50% men), aged 31–55 years, in ISO 8589-compliant sensory booths. Each panelist rated color, aroma, texture, flavor, and overall taste using a nine-point hedonic scale. Water and crackers were provided for palate cleansing between samples. Additionally, a consumer test was conducted with 10 children aged 7–12 years (5 boys, 5 girls), all of whom provided assent prior to participation in the study. For the children’s consumer test, written informed consent was obtained from parents or legal guardians, and assent was obtained from all participants.

### 2.8 Statistical analysis

Data were expressed as the mean ± standard deviation of triplicate experiments. Statistical analyses included one-way ANOVA, followed by Tukey’s HSD test (α = 0.05) using IBM SPSS v25. Correlations between physicochemical properties and sensory scores were evaluated using Pearson’s correlation coefficient.

### 2.9 Ethical considerations

The study was approved by the Human Research Ethics Committee of the Escuela Superior Politécnica de Chimborazo (Code OI-02-CEISH-ESPOCH-2023). Written informed consent was obtained from all adult participants. For children, parental/legal-guardian consent and child assent were obtained prior to participation.

## 3 Results

The formulation with the best texture was Formula 3, which underwent an extrusion process at a temperature range of 80/110/135 °C. The pressure was adjusted to obtain a crunchy texture, resulting in flakes with the desired shape and size. The flakes were then placed in an oven at 65°C. To prevent moisture formation and ensure product stability, the flakes were allowed to cool at room temperature.

It is important to note that both the Formula 3 mixture and the final product were subjected to quality analysis and control, as detailed below.

### 3.1 Proximate quality of breadfruit flour

The proximate analysis of breadfruit flour showed an average moisture content of 10.8%, protein content of 10.12%, and fiber content of 4.06% (Table 2). These values are within the nutritional targets for cereal ingredients. However, the ash content (1.57%) and fat content (2.45%) slightly exceeded the upper limits set by INEN 616:2015 for wheat flour (0.8 and 2.0%, respectively). The higher mineral fraction can be attributed to the volcanic soils of coastal Ecuador, though formulation studies should monitor ash-driven flavor changes in the final product.

**TABLE 2:** Physicochemical and microbiological characteristics of breadfruit flour.

Parameter	Unit	R1	R2	R3	Mean	Standard/Method
Moisture	%	11.1	10.5	10.8	10.8	NTE INEN 540
Ash	%	1.57	1.56	1.58	1.57	NTE INEN 544
Protein	%	10.12	10.15	10.10	10.12	NTE INEN 543
Fiber	%	4.06	4.12	4.00	4.06	NTE INEN 522
Fat	%	2.46	2.50	2.38	2.45	NTE INEN 523
Mesophilic aerobes	CFU/g	1 × 10^2^	1 × 10^2^	1 × 10^2^	1 × 10^2^	NTE INEN 540
Total coliforms	CFU/g	12	10	12	11	NTE INEN 544
Escherichia coli	CFU/g	0	0	0	0	NTE INEN 543
Molds and yeasts	CFU/g	3	1	3	2	NTE INEN 523

R1, R2, and R3 refer to replicate measurements. CFU: Colony-Forming Units. NTE INEN: Ecuadorian technical standards of the Ecuadorian Institute of Standardization.

### 3.2 Microbiological safety

Microbiological analysis confirmed that all microbial counts were well below Ecuadorian statutory limits (Table 2). The mesophilic aerobes averaged 1 × 10^2^ CFU/g, total coliforms were 11 MPN/g, and E. coli was undetected ( < 10 CFU/g). Molds and yeasts were present at 2 CFU/g. These results confirm that blanching and low-temperature drying produce a shelf-stable, safe flour that meets the required microbiological safety standards.

### 3.3 Chemical contaminants

The levels of lead (Pb) and cadmium (Cd) were found to be well below the regulatory limits. Specifically, Pb was present at 0.085 mg/kg and Cd at 0.012 mg/kg, both of which are within the acceptable limits set by INEN 616 (0.2 mg/kg for each) (Table 3). Aflatoxins (B_1_, B_2_, G_1_, G_2_) were not detected, with a LOD of 0.5 μg/kg, indicating proper handling of raw materials and compliance with safety standards.

**TABLE 3:** Heavy metal content and fatty-acid profile of breadfruit flour.

Analyte/fraction	Unit	Value	INEN limit	Compliance	Standard/reference
Cadmium (Cd)	mg/kg	0.012	0.2 (INEN 616)	Within limit	INEN 616
Lead (Pb)	mg/kg	0.085	0.2 (INEN 616)	Within limit	INEN 616
Saturated (S)	%	25.1	—	—	—
Monounsaturated (M)	%	11.9	—	—	—
Polyunsaturated (P)	%	52.8	—	—	—
Unidentified (R)	%	10.3	—	—	—

INEN 616 is the Ecuadorian technical regulation that sets maximum levels of cadmium and lead in foods (0.2 mg/kg each). Reported heavy-metal values are below the regulatory limits. Fatty-acid fractions are expressed as percentage of total fatty acids; “Unidentified (R)” comprises minor unresolved peaks. “—” indicates not applicable. Analyses were carried out on Formula 3.

### 3.4 Lipid class distribution and bioactive compounds

The breadfruit flour exhibited a high content of polyunsaturated fatty acids (PUFA), which accounted for 52.8% of the total lipids. The remaining lipid content was composed of saturated fatty acids (25.1%) and monounsaturated fatty acids (11.9%) (Table 3). The bioactive compounds in the flour included 298.7 mg GAE/100 g total phenolics and 41.4 mg β-carotene/100 g carotenoids, highlighting the flour’s potential as a source of antioxidants relative to other tropical starches.

### 3.5 Compliance with physicochemical standards

When benchmarked against INEN 616, the breadfruit flour met the moisture and protein specifications but marginally exceeded the limits for ash and fat content. Given the absence of gluten in breadfruit, direct comparisons with wheat standards should be interpreted cautiously. Nevertheless, fat reduction through solvent-assisted deoiling could be explored for applications where lipid oxidation is a concern.

### 3.6 Extruded corn breadfruit flakes

Using a constant 20.70% breadfruit flour, varying sugar, flavor, and colorant levels produced well-expanded flakes across formulations (Table 4). F3 exhibited the highest expansion (4.0 ± 0.1), greater hardness (14.7 ± 0.4 N), and the best overall liking (7.9 ± 0.33), with differences supported by Tukey groupings (*p* < 0.05). Moisture of F1–F3 was 11.1, 10.5, and 10.8%, respectively.

**TABLE 4:** Physicochemical and sensory parameters (mean ± SD, n = 3) with Tukey letters.

Parameter	Formula 1 (F1)	Letters	Formula 2 (F2)	Letters	Formula 3 (F3)	Letters
Color L	71.7 ± 1.5	C	69.6 ± 0.7	b	67.4 ± 0.7	A
Expansion	3.6 ± 0.1	C	3.8 ± 0.1	b	4.0 ± 0.1	A
Hardness (N)	11.9 ± 0.2	c	12.7 ± 0.4	b	14.7 ± 0.4	A
Moisture (%)	11.1 ± 0.3	a	10.5 ± 0.2	b	10.8 ± 0.1	A
Water activity (a_*v*_)	0.28 ± 0.01	a	0.27 ± 0.02	a	0.26 ± 0.01	A
Bulk density (g/cmł)	0.29 ± 0.01	b	0.31 ± 0.01	a	0.30 ± 0.01	Ab
Sensory score	6.2 ± 0.3	b	6.2 ± 0.1	b	7.9 ± 0.3	A

Different letters indicate significant differences at *p* < 0.05 (Tukey’s HSD).

### 3.7 Statistical appraisal of extrusion performance

The statistical analysis of the extrusion process revealed consistent effects of formulation variables on the physical and sensory properties of the extruded flakes. Since the proportion of breadfruit flour was fixed at 20.70% across all treatments, observed differences were mainly attributed to the varying levels of sugar, flavoring, and colorant used in the formulations (Table 4).

The expansion ratio ranged from 3.60 ± 0.05 to 4.02 ± 0.02, with formulation F3 exhibiting the highest value, indicating better puffing capacity and structural uniformity (*p* < 0.05). Texture analysis showed that hardness increased proportionally with higher sugar content and decreased expansion. F3, which combined moderate sugar levels and balanced extrusion parameters, exhibited a hardness of 14.7 ± 0.4 N, representing an optimal balance between crispness and mechanical strength.

Moisture content remained stable across all treatments (10.5–11.1%), slightly above the < 10% limit recommended for ready-to-eat cereals. Nevertheless, this did not adversely affect flake quality or sensory evaluation. Among the tested formulations, F3 achieved the highest sensory score (7.9 ± 0.3 on the nine-point scale), significantly outperforming F1 and F2 (*p* < 0.05).

In summary, maintaining a constant level of 20.70% breadfruit flour while optimizing other formulation variables yielded extrudates with desirable physical characteristics and high consumer acceptability. Formulation F3 offered the best balance of expansion, texture, and appearance, confirming the potential of breadfruit flour as a functional ingredient in extruded breakfast cereals.

## 4 Discussion

The bromatological characterization of breadfruit flour (Artocarpus altilis) revealed its promising properties as a functional ingredient in the food industry. The fiber content (4.06%) supports its role in reducing chronic low-grade inflammation (CLGI), which is associated with the early onset of cardiometabolic risk factors, cholesterol reduction, glycemic control, and prevention of constipation, among other gastrointestinal benefits such as aiding in the prevention of colon cancer and diverticular disease (11–13). Moreover, the fiber content has been linked to alterations in the intestinal microbiome, which plays a crucial role in functional gastrointestinal disorders such as irritable bowel syndrome (14). The complex interplay between gut microbiota and host health, particularly in conditions like IBS, underscores the potential of dietary fibers to modulate microbial activity and produce beneficial metabolites like short-chain fatty acids (15, 16). Beyond the direct benefits of fiber, phenolic compounds found in cereals, including potentially in breadfruit, also contribute to gut health by acting as prebiotics, fostering beneficial bacterial taxa while suppressing harmful ones (15). These health benefits are well-documented in studies on fiber-rich foods. Additionally, the carbohydrate content (71%) makes breadfruit flour an excellent energy source, which is particularly beneficial for inclusion in diets of vulnerable populations where food security is critical

Extrusion processing is essential in modifying the functional properties of breadfruit flour. Studies have demonstrated that changes in extrusion conditions, such as temperature and feed moisture, significantly impact the swelling power, solubility, and cold viscosity of breadfruit flour (2). These changes suggest that processing disrupts or rearranges starch and protein structures, leading to improved gelatinization and water-holding capacity. In line with this, thermal and rheological analyses have shown that gelatinization of breadfruit starch occurs around 75 °C under high-moisture conditions (17), while low-moisture conditions lead to the formation of additional endothermic peaks, linked to the melting of amylose-lipid complexes. Moreover, the retrogradation of starch decreases paste viscosity, potentially influencing both the texture and digestibility of the final product.

The fatty acid profile analysis revealed a dominance of polyunsaturated fatty acids (PUFAs), which constitute 52.8% of the total lipids. These fatty acids are well known for their cardiovascular benefits, particularly their ability to lower LDL cholesterol and improve vascular health (18). Long-chain polyunsaturated fatty acids (LC-PUFAs), especially DHA, play a crucial role in the development and function of the brain and central nervous system (19), with strong associations between infant DHA status and neurodevelopmental outcomes. Studies indicate that n-3 PUFA supplementation leads to improvements in mental development indices (MDI) (20), particularly in infants, and enhances language skills, especially when supplementation begins during the maternal period. These fatty acids also correlate with better cognitive and motor function.

In addition to fatty acids, breadfruit flour is rich in total phenolic compounds (298.7 mg GAE/100 g), including p-coumaric acid, vanillic acid, and quercetin, which collectively make up 57.9% of the identified polyphenols. These compounds are recognized for their potent antioxidant and anti-inflammatory activities (21), contributing to the flour’s overall health-promoting properties (22, 23). These phenolic acids and flavonoids, notably p-coumaric acid, vanillic acid, and quercetin, contribute to scavenging free radicals through mechanisms such as hydrogen atom transfer and single electron transfer, largely attributed to their hydroxyl substituents (21). They also chelate metal ions like ferrous ion by forming complexes through their carbonyl and hydroxyl groups, which prevents the initiation of further oxidative reactions (24–26). This dual action effectively reduces oxidative damage by mitigating reactive oxygen species formation and modulating pathways such as the Nrf2-ARE pathway, which is crucial for cellular defense against oxidative stress and inflammation (27–29). Furthermore, the presence of specific phenolic compounds such as those found in breadfruit flour, including p-coumaric acid, vanillic acid, and quercetin, has been shown to inhibit key enzymes involved in carbohydrate metabolism, such as α-amylase and α-glucosidase, thereby contributing to glycemic control (30).

These phenolic compounds are known for their immunomodulatory and antimicrobial properties. For instance, quercetin has shown potential in inhibiting rotavirus protein expression, viral replication, and infectious particle production *in vitro* (31). The antiviral effects of quercetin have also been observed against several other viral infections, including influenza and rhinovirus (32). Additionally, these compounds exhibit notable antioxidant properties, suggesting their potential use in preventing oxidative stress, which is a major factor in chronic diseases.

The sensory evaluation of breadfruit-corn flakes with 20.7% breadfruit flour showed high consumer acceptability. The combination of breadfruit flour and corn helped balance the nutritional profile while enhancing the sensory properties, making the product suitable as a functional snack. This aligns with the current demand for healthier, appealing products that maintain adequate caloric and nutritional value.

Processing and packaging determine shelf-life and quality. In this study, flake moisture ranged from 10.5 to 11.1% (Table 4), slightly above the < 10% often recommended for ready-to-eat cereals; therefore, a short post-drying step and moisture-barrier packaging are advisable to enhance stability. The products complied with national microbiological criteria (e.g., NTE INEN 2561:2010; NTE INEN 540:2010), supporting their safety for consumption. Implementing moisture-barrier, metallized pouches and, where appropriate, oxygen absorbers could further preserve texture and bioactive compounds during storage. According to studies on packaging technologies, the application of Modified Atmosphere Packaging (MAP) has proven to extend the shelf life of various food products, including strawberries, by controlling the gaseous environment (CO_2_, O_2_, and N_2_) (33). This reduces the respiration rate, enzymatic deterioration, and microbial proliferation, thereby preserving the nutritional value and phytochemical stability of the products. Implementing MAP and other preservation techniques could significantly improve the storage stability of breadfruit-based products.

In line with this, optimizing drying and packaging conditions is essential for ensuring a longer shelf life. Studies have highlighted that these adjustments are crucial for extruded products, where shelf-life extension and the preservation of bioactive compounds can directly impact product availability and consumer acceptance. Furthermore, the microbiological analysis in this study confirmed that the breadfruit flakes met the microbiological standards set by INEN 616:2015 (34), validating that the product is safe for human consumption.

Overall, both breadfruit flour and breadfruit-corn flakes stand out as innovative and sustainable alternatives for the Ecuadorian food industry, particularly as the formal production of breadfruit is not yet established, and its consumption remains largely undervalued. Given its sustainability and respect for local food cultures, breadfruit flour could gain widespread acceptance and become popular in other regions as well.

Child undernutrition remains a global concern, and is a multifactorial issue that hampers children’s growth, cognitive development, and immune health. It stems not only from food insecurity but also from low educational levels, limited healthcare access, and inadequate complementary feeding practices during early childhood. The food industry has a key role in developing nutrient-dense products, rich in essential micronutrients like iron, zinc, vitamin A, and folic acid, which are crucial for preventing childhood deficiencies. To be effective, these products must be organoleptically appealing, easily integrated into children’s diets, and accessible to vulnerable populations. Recent studies show that fortified foods, specifically designed for children, can improve nutritional biomarkers without sacrificing sensory acceptability (35). Thus, collaboration between the public and private sectors is vital to promote scientific-based food innovations and contribute to sustainable strategies aimed at combating child undernutrition. Breadfruit-based products not only diversify functional foods but also contribute to the valorization of underutilized crops like breadfruit, thereby fostering sustainability and food security.

## 5 Conclusion

Breadfruit flour stands out for its high content of fiber, carbohydrates, and phenolic compounds, which enhances its antioxidant properties and its ability to support digestive and cardiovascular health. These characteristics make it a promising ingredient for the development of food products aimed at combating malnutrition and promoting health in vulnerable populations. On the other hand, flakes formulated F3 with 20.7% breadfruit flour demonstrated high sensory acceptability and met microbiological standards, validating their viability as a functional and safe snack. However, the slightly elevated moisture content highlights the need to optimize drying and storage processes to improve product stability.

Overall, the results confirm the potential of breadfruit as an underutilized food resource that can be harnessed to diversify the functional food market in Ecuador. Its development not only fosters food innovation but also promotes the valorization of local crops, contributing to national sustainability and food security. Future research should focus on optimizing production processes and exploring additional applications across other segments of the food industry.

## Data Availability

The raw data supporting the conclusions of this article will be made available by the authors, without undue reservation.
