# Correction: Production and characterization of breadfruit flour (*Artocarpus altilis*): an alternative for the elaboration of flakes for children's alimentation

**DOI:** 10.3389/fnut.2026.1799573

**Published:** 2026-02-19

**Authors:** Cristina Arteaga, Ruth Arias-Gutiérrez, Adriana Rodríguez, Susana Heredia-Aguirre, Mónica Gozalbo, Jesús Blesa, Renata Alejandra Alvarado-Barba, Cristina Gabriela Ríos-Romero, Dayana Villavicencio Barriga, Jessenia Vásquez, Karol Egas, Elizabeth Quiroga-Torres, Alberto Bustillos, Tannia Valeria Carpio-Arias

**Affiliations:** 1 Carrera de Nutrición y Dietética, Facultad de Ciencias de la Salud, Universidad Técnica de Ambato, Ambato, Ecuador; 2 Facultad de Ciencias de la Vida, Universidad Estatal Amazónica, Puyo, Ecuador; 3 Faculty of Sciences, and Pharmacy Program, Riobamba-Chimborazo, Escuela Superior Politécnica de Chimborazo, Riobamba, Ecuador; 4 Carrera de Nutrición y Dietética, Facultad de Salud Pública, Escuela Superior Politécnica de Chimborazo, Riobamba, Ecuador; 5 Department of Preventive Medicine and Public Health, Food Sciences, Toxicology and Legal Medicine, Universitat de València, Valencia, Spain; 6 Sede Orellana, Escuela Superior Politécnica de Chimborazo, Riobamba, Ecuador; 7 Research Group on Human Food and Nutrition (GIANH), Riobamba-Chimborazo, Escuela Superior Politécnica de Chimborazo, Riobamba, Ecuador; 8 Ecuador Kuskiykuy Academic and Scientific Research Group in Biomedical Sciences with Social Projection, Yachay Suntur, Technical University of Ambato, Ambato, Ecuador; 9 Laboratorio de Bioquímica, Carrera de Agronomía, Facultad de Ciencias Agropecuarias, Universidad Técnica de Ambato, Ambato, Ecuador

**Keywords:** breadfruit flour, extrusion cooking, breakfast cereal flakes, sensory acceptability, food safety

In the published article, there was an error in the affiliations assigned to the authors. The author, Renata Alejandra Alvarado-Barba, was erroneously assigned to affiliation 5, “Department of Preventive Medicine and Public Health, Food Sciences, Toxicology and Legal Medicine, Universitat de València, Valencia, Spain” and affiliation 6, “Sede Orellana, Escuela Superior Politécnica de Chimborazo, Riobamba, Ecuador”. Their correct affiliations should be affiliation 6, “Sede Orellana, Escuela Superior Politécnica de Chimborazo, Riobamba, Ecuador”, and affiliation 7, “Research Group on Human Food and Nutrition (GIANH), Riobamba-Chimborazo, Escuela Superior Politécnica de Chimborazo, Riobamba, Ecuador.”

The author, Cristina Gabriela Ríos-Romero, was erroneously assigned to affiliation 6, “Sede Orellana, Escuela Superior Politécnica de Chimborazo, Riobamba, Ecuador”. Their correct affiliation should be affiliation 7, “Research Group on Human Food and Nutrition (GIANH), Riobamba-Chimborazo, Escuela Superior Politécnica de Chimborazo, Riobamba, Ecuador”.

The author, Dayana Villavicencio Barriga, was erroneously assigned to affiliation 6, “Sede Orellana, Escuela Superior Politécnica de Chimborazo, Riobamba, Ecuador”. Their correct affiliation should be affiliation 7, “Research Group on Human Food and Nutrition (GIANH), Riobamba-Chimborazo, Escuela Superior Politécnica de Chimborazo, Riobamba, Ecuador”.

The author, Elizabeth Quiroga-Torres, was erroneously assigned to affiliation 7, “Research Group on Human Food and Nutrition (GIANH), Riobamba-Chimborazo, Escuela Superior Politécnica de Chimborazo, Riobamba, Ecuador”. Their correct affiliation should be affiliation 8, “Ecuador Kuskiykuy Academic and Scientific Research Group in Biomedical Sciences with Social Projection, Yachay Suntur, Technical University of Ambato, Ambato, Ecuador”.

The author, Alberto Bustillos, was erroneously assigned to affiliation 8, “Ecuador Kuskiykuy Academic and Scientific Research Group in Biomedical Sciences with Social Projection, Yachay Suntur, Technical University of Ambato, Ambato, Ecuador”. Their correct affiliation should be affiliation 9, “Laboratorio de Bioquímica, Carrera de Agronomía, Facultad de Ciencias Agropecuarias, Universidad Técnica de Ambato, Ambato, Ecuador.”

The author, Tannia Valeria Carpio-Arias, was erroneously assigned to affiliation 6, “Sede Orellana, Escuela Superior Politécnica de Chimborazo, Riobamba, Ecuador”. Their correct affiliation should be affiliation 7, “Research Group on Human Food and Nutrition (GIANH), Riobamba-Chimborazo, Escuela Superior Politécnica de Chimborazo, Riobamba, Ecuador”.

The original version of this article has been updated.

